# Crosstalk between autophagy and epithelial-mesenchymal transition and its application in cancer therapy

**DOI:** 10.1186/s12943-019-1030-2

**Published:** 2019-05-24

**Authors:** Hong-Tao Chen, Hao Liu, Min-Jie Mao, Yuan Tan, Xiang-Qiong Mo, Xiao-Jun Meng, Meng-Ting Cao, Chu-Yu Zhong, Yan Liu, Hong Shan, Guan-Min Jiang

**Affiliations:** 1 Department of Clinical Laboratory, The Fifth Affiliated Hospital of Sun Yat-sen University, Zhuhai, 2528000 Guangdong China; 2 Cancer Hospital and Cancer Research Institute, Guangzhou Medical University, Guangzhou, Guangdong China; 3 Department of Laboratory Medicine, State Key Laboratory of Oncology in South China, Collaborative Innovation Center for Cancer Medicine, Sun Yat-sen University Cancer Center, Guangzhou, Guangdong China; 4 Department of Clinical Laboratory, Hunan Cancer Hospital, The Affiliated Cancer Hospital of Xiangya School of Medicine, Central South University, Changsha, Hunan China; 5 Department of Gastrointestinal Surgery, The Fifth Affiliated Hospital of Sun Yat-sen University, Zhuhai, Guangdong China; 6 Department of Endocrinology, The Fifth Affiliated Hospital of Sun Yat-sen University, Zhuhai, Guangdong China; 7 Department of Clinical Laboratory, The First Affiliated Hospital of University of South China, Hengyang, Hunan China; 8 Department of Geriatrics, The Fifth Affiliated Hospital of Sun Yat-sen University, Zhuhai, Guangdong China; 9 Guangdong Provincial Key Laboratory of Biomedical Imaging, The Fifth Affiliated Hospital of Sun Yat-sen University, Zhuhai, 2528000 Guangdong China

**Keywords:** Autophagy, Epithelial-mesenchymal transition, Cancer metastasis, Anticancer therapy

## Abstract

Autophagy is a highly conserved catabolic process that mediates degradation of pernicious or dysfunctional cellular components, such as invasive pathogens, senescent proteins, and organelles. It can promote or suppress tumor development, so it is a “double-edged sword” in tumors that depends on the cell and tissue types and the stages of tumor. The epithelial-mesenchymal transition (EMT) is a complex biological trans-differentiation process that allows epithelial cells to transiently obtain mesenchymal features, including motility and metastatic potential. EMT is considered as an important contributor to the invasion and metastasis of cancers. Thus, clarifying the crosstalk between autophagy and EMT will provide novel targets for cancer therapy. It was reported that EMT-related signal pathways have an impact on autophagy; conversely, autophagy activation can suppress or strengthen EMT by regulating various signaling pathways. On one hand, autophagy activation provides energy and basic nutrients for EMT during metastatic spreading, which assists cells to survive in stressful environmental and intracellular conditions. On the other hand, autophagy, acting as a cancer-suppressive function, is inclined to hinder metastasis by selectively down-regulating critical transcription factors of EMT in the early phases. Therefore, the inhibition of EMT by autophagy inhibitors or activators might be a novel strategy that provides thought and enlightenment for the treatment of cancer. In this article, we discuss in detail the role of autophagy and EMT in the development of cancers, the regulatory mechanisms between autophagy and EMT, the effects of autophagy inhibition or activation on EMT, and the potential applications in anticancer therapy.

## Background

Autophagy can be stimulated by intracellular or environmental stresses, including nutrient deprivation, hypoxia, and damaged organelles. Generally, the complete macroautophagic process is divided into the following stages: induction, vesicle nucleation, vesicle elongation, docking and fusion, degradation, and recycling. The degraded and recycled metabolites can provide energy supplies and basic nutrients for cells growth [1]. Recent observations have shown that autophagy can suppress cancer development by eliminating potentially harmful components and mutant DNA and chromosomes or can promote cancer development by overcoming the stressful conditions and producing nutrients and adenosine triphosphate (ATP) to maintain protein synthesis and other metabolic functions, which depends on the cell/tissue types and the stages of cancer [2]. Thus, the effects of autophagy on anticancer treatment remain to be investigated in depth.

It is well-known that the epithelial-mesenchymal transition (EMT) is considered to be a major driver of cancer exacerbation from initiation to metastasis and plays a key part in the induction of cancer progression, metastasis, and drug resistance [3, 4]. The process of EMT contains adhesion junctions and loss of substrate polarity; acquisition of mesenchymal characteristics, such as spindle-shaped cell morphology and reorganization of actin stress fibers; enhancement of movement; and invasion and resistance to apoptosis [5].

As is well known, autophagy and EMT are major biological processes in the occurrence and development of cancer, and there is a complex relationship between autophagy-correlated and EMT-correlated signaling pathways. In previous studies, it has been found that EMT-related signaling pathways can trigger or repress autophagy. Significantly, autophagy is also involved in the induction and inhibition of EMT. On the one hand, EMT requires autophagy to support the viability of potentially metastasis of cancer cells. It has been indicated that an EMT-like phenotype corresponds to a higher autophagy flux, and the combination of an autophagy inhibitor (chloroquine) with the current therapeutic regimen could be more beneficial alongside the repressed EMT in renal cell carcinoma (RCC) [6]. On the other hand, a growing body of additional evidence indicates that autophagy acts to prevent EMT, and the activation of the autophagy may abate the acquisition of the EMT phenotype in cancer cells. It has been shown that induction of autophagy by nutrient deprivation or mechanistic target of rapamycin (mTOR) pathway inhibition leads to reduced migration and invasion in glioblastoma cells. Autophagy impairment determined by silencing of autophagy-related genes 5 (ATG5), ATG7, or Beclin-1 results in an increment of cell motility and invasiveness with the up-regulation of SNAIL and SLUG, two of the major transcription factors of the EMT process [7]. Because of the dual effects of autophagy on EMT, inhibiting EMT by targeting autophagy might be a novel strategy for anticancer therapy.

Some studies have demonstrated the effect of preclinical application of autophagy inhibitors or activators on anticancer treatment by regulating EMT. Collectively, in this review, we discuss in detail the role of autophagy and EMT in the development of cancers, the regulatory mechanisms between autophagy and EMT, the effects of autophagy inhibition or activation on EMT, and the potential applications in anticancer therapy.

## The role of autophagy and EMT in the development of cancer

Autophagy is viewed as type II programmed cell death, namely, autophagic cell death, stimulated by cellular or environmental stresses in order to clear senescent organelles, protein aggregates, and intracellular pathogens through the formation of autophagosomes, subsequently targeting to lysosomal digestion, which maintains a steady state for cell survival by engulfing the metabolic waste, inhibiting the production of reactive oxygen species (ROS), eliminating damaged mitochondria and peroxisomes, and reducing DNA damage and chromosomal instability.

The macroautophagic process is triggered by the formation of the Atg1/ULK complex when the induction signals suppress mammalian target of rapamycin complex 1 (mTORC1). Then, the ULK complex binding to the phosphatidylinositol 3-kinase (PI3K) complex (Beclin1–hVps34–PI3K) establishes a putative mammalian preautophagosomal structure, probably together with vacuole membrane protein 1 (VMP1) and Atg9, where PI3K locally generates PI3P. Next, phagophore elongation bases on the Atg5-Atg12 and the microtubule-associated light chain 3 (MAP-LC3/Atg8/LC3) conjugation systems. Phagophore progressively engulfs a portion of the cytoplasm to form the double-membrane autophagosome after elongation and fusion. Finally, the fusion of an autophagosome with a lysosome triggers the formation of an autolysosome and degradation of the loads, and the recycles are released back into the cytosol for reuse [8, 9].

Normally, autophagy can prevent cancer initiation by removing intracellular mutants, damaged mitochondria, infectious pathogens, and misfolded proteins and inhibiting inflammation. However, during the advanced stages of cancer, autophagy promotes cancer development by producing nutrient substances and releasing ATP, which is beneficial for boosting a variety of biological metabolisms and satisfies the high demand for cancer cell proliferation, invasion, and metastasis [10]. Therefore, autophagy might exact the opposite effects on anticancer treatment in different tissue or development stages of cancer [11].

It was reported that autophagy not only can decrease the apoptosis of isolated cancer cells but also can inhibit the apoptosis of cancer cells in vivo during metastasis by regulating the cancer microenvironment, which indicates that autophagy plays an important role in promoting cancer metastasis, and the inhibition of autophagy might be an effective treatment strategy for malignant cancer [12]. According to current research, autophagy inhibition has been applied to a variety of cancer therapies, such as glioma, myeloma, breast cancer, rectal cancer, and prostate cancer [6]. As a “double-edged sword” in cancer, the regulatory mechanism of autophagy in cancer is complicated, but the observations about specific molecular markers of autophagy have attracted great attention in terms of current anticancer treatment. The choice of autophagy inhibitors and agonists during treatment deserves to be explored more deeply.

EMT is a dynamic multistep process that involves the loss of intercellular adhesion, the destruction of the cancer basement membrane and extracellular matrix, the reconstruction of the cytoskeleton, and the enhancement of cell motility [13, 14], which increased the difficulty of cancer treatment in clinical. There are three types of EMT that are based on the specific biological environment. Type 1 EMT is mainly related to embryo implantation, development, and organogenesis. Type 2 EMT is mainly associated with injury repair, tissue regeneration, and organ fibrosis. Type 3 EMT refers to phenotypic transformation associated with epithelial cell malignancy, which facilitates metastatic cancer cells to maintain a certain epithelial character while obtaining a mesenchymal phenotype, so EMT involving cancer metastasis refers to type 3.

EMT implies a complete transdifferentiation from a functional epithelial cell into a mesenchymal-like cell, which occurs along with the inhibition of senescence and anoikis as well as acquisition of immunosuppression and cancer stem cell (CSC)–like features, resistance to anticancer drugs, and apoptosis. These processes involve multiple signal transduction pathways and complicated molecular mechanisms [3]. Naturally, EMT is regulated by exosomes, extracellular matrix, oxygen deficit, and soluble factors such as hepatocyte growth factor, fibroblast growth factor (FGF), and members of transforming growth factor β (TGF-β) [15]. E-cadherin is essential for calcium-dependent cell-cell adhesion and signal transduction; its decrease or loss acts as a crucial role in EMT induction by promoting cell invasive movement and diffusion [16]. In addition, E-cadherin dysfunction is reflected by gene mutation, which will result in abnormal protein synthesis and hydrolysis.

During the EMT process, Zinc-finger E-box binding homeobox 1 (ZEB1) and SNAIL are regarded as the main EMT transcription factors to initialize and maintain the EMT process. ZEB1 is one of the most critical EMT conversion molecules, as a zinc finger structure of the DNA binding protein, which can prohibit E-cad gene expression by binding to E-cad promoter site. Furthermore, ZEB1 expression in human bronchial epithelial cells directly repressed epithelial splicing regulatory protein 1 (ESRP1), leading to increased expression of a mesenchymal splice variant of CD44 and increment of EMT in lung cancer [17]. SNAIL is regulated by various signals from the cancer microenvironment, is a prominent inducer of EMT, strongly repressing E-cadherin expression [18]. Mechanistically, in cholangiocarcinoma, atypical protein kinase C-iota (aPKC-ι) directly phosphorylates specificity protein 1 (Sp1) to up-regulate P-Sp1 that increased SNAIL expression by promoting Sp1 binding to the SNAIL promoter, resulting in EMT changes and immunosuppression [19].

It has been confirmed that EMT is a crucial step in the migration of cancer cells, which is controlled by different signaling transduction pathways and networks, such as autocrine IL11/IL6-gp130/JAK2/STAT, fibronectin-integrin, GAS6-Axl/Tyro3, PDGFR/FGFR/RET, and TGF-β R networks [20]. The current therapy strategies and drug development can focus on targeting these signal transduction pathways. However, there is a biological phenomenon of cancer cell dormancy when cancer enters into the stage of aggressive metastasis, which facilitates target cells to escape chemotherapy and radiotherapy, eventually leading to low survival rate and high cancer recurrence. Therefore, to find the best treatment period, further understanding the specific regulation mechanism between EMT and cancer metastasis is urgently needed.

## Crosstalk between autophagy and EMT

Autophagy is mainly controlled by PI3K/AKT/mTOR, Beclin-1, p53, and JAK/STAT signaling pathways. These autophagy-related regulatory pathways have a dramatic impact on EMT. In the process of EMT, there are several signaling pathways, including integrin, WNTs, NF-kB, and TGF-β signaling pathways, that play a crucial role in autophagy. Furthermore, increasing observations have indicated that the functional interaction between cytoskeleton and mitochondria is also the crucial regulatory mechanism in the process of autophagy and EMT.

### The PI3K/AKT/mTOR signaling pathway

**Fig. 1:**
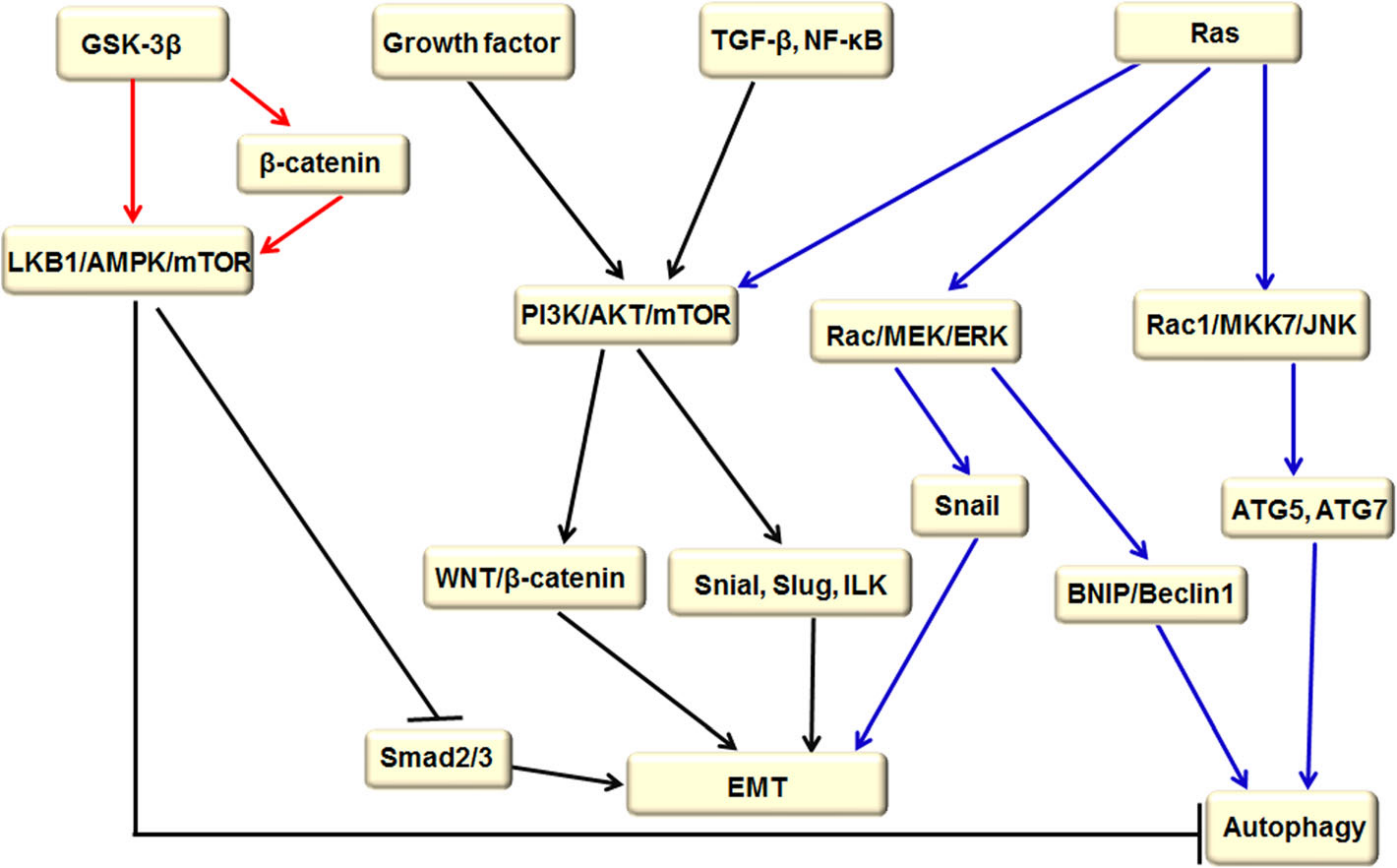
mTOR signaling pathway regulated autophagy and EMT. PI3K activation is induced by interaction with a growth factor receptor, direct binding to Ras, also induced by NF-κB and TGF-β activation. Activation of the PI3K/AKT signaling pathway blocks autophagy by prohibiting mTOR. The PI3K/Akt pathway positively regulates WNT/β-catenin through phosphorylating the serine at residue 552 in β-catenin and the serine at residue 9 in glycogen synthase kinase 3β (GSK3β), which increases intracellular β-catenin levels that combine with E-cadherin to promote EMT. Moreover, The PI3K/Akt pathway activity up-regulates nuclear factors SNAIL and SLUG, contributing to EMT activation. GSK-3β directly induces autophagy by activating LKB1/AMPK and in turn prohibiting the PI3K/AKT/mTOR pathway. It also indirectly triggers autophagy through promoting the hydrolysis of β-catenin protein. LKB1/AMPK activation plays a critical role in stimulating autophagy via decreasing the ratio of p-mTOR/mTOR and p-p70s6k/p70s6k. In addition, LKB1/AMPK hinders EMT by inhibiting Smad2/3 and TGF-β activity. Ras protein mutation not only activates the Ras/Rac1/Mkk7/JNK pathway (with JNK in turn binding to Atg5/Atg7) but also induces the Ras/Raf1/MEK1/2/ERK signaling pathway, which results in autophagy activation and EMT enhancement

mTOR is a serine-threonine kinase that controls several important aspects of mammalian cell function. Changes in mTOR activity have a dramatic impact on rates of translation, transcription, protein degradation, cytoskeleton dynamics, cell metabolism, and autophagy [21]. However, the upstream and downstream signal transduction pathways of mTOR are accurately complicated. The predominant upstream pathway is PI3K/AKT/mTOR that is significantly regulated by LKB1/adenosine monophosphate–activated protein kinase (AMPK) and Ras signaling pathways [22–24]. There are two main mTOR downstream signaling pathways. First, when mTOR is activated by various stresses, phosphorylated ribosomal protein S6 (P70S6) will promote mRNA translation, adhesion of ribosomes, and endoplasmic reticulum, inhibiting the delivery of endoplasmic reticulum and the formation of autophagic membrane [25]. Second, mTOR activity also inhibits the activity of eukaryotic initiation factor 4E (eIF4E)-binding proteins (4E-BPs) by phosphorylating 4E-BPs and releasing eIF4E [26]. Significantly, autophagy activation by inhibiting the mTOR pathway attenuates migration and invasion of gallbladder cancer via EMT inhibition [27]. For example, metformin can suppress the proliferation, migration, and EMT by inhibiting mTOR signaling and stimulating autophagy in thyroid cancer cell lines [28]. Likewise, water stress proteins (WSP1)-induced autophagy through down-regulating PI3K/AKT/mTOR pathway could degrade β catenin and inhibit EMT through increasing E-cadherin and decreasing N-cadherin, which inhibits cancer migration [29] (Fig. 1).

PI3Ks activity can lead to cancer cell migration, adhesion, and malignant transformation, together with degradation of the extracellular matrix, which prohibit autophagy and enhance EMT by up-regulating SNAIL, SLUG, integrin-linked kinase (ILK), and WNT/β-catenin signaling. PI3K is activated by the interaction with a growth factor receptor or connexin with a phosphorylated tyrosine residue, causing a dimer conformational change. In addition, PI3K activation is induced by direct binding to Ras or by nuclear transcription factor κB (NF-κB) and TGF-β activation.

Activation of the PI3K/AKT/mTOR signaling pathway not only blocks autophagy but also plays an important role in regulating EMT [30]. It has been found that activation of the PI3K/AKT/mTOR signaling pathway can lead to EMT changes in tongue squamous cell carcinoma. Interestingly, P13K activation can facilitate the increment of ILK. ILK is a serine-threonine protein kinase that binds to the β-integrin cytoplasmic domain and down-regulates E-cadherin, leading to the EMT [31]. Moreover, the PI3K/Akt pathway positively regulates WNT/β-catenin through phosphorylating the serine at residue 552 in β-catenin and the serine at residue 9 in glycogen synthase kinase 3β (GSK3β), which increases levels of intracellular β-catenin that combines with E-cadherin to promote EMT [32]. In addition, the PI3K/AKT/mTOR signaling pathway is induced by growth factor, contributing to the promotion of cell metastasis and EMT by up-regulating nuclear factor, SNAIL, and SLUG and promoting matrix metalloproteinase (MMP) to degrade the cell matrix, which synergies with other EMT signaling pathways. For instance, TGF-β induces EMT by directly or indirectly activating the PI3K/Akt signaling pathway [33]. Invasion, migration, and TGF-β–induced EMT would be suppressed through the inhibition of the PI3K/Akt/mTOR signaling pathway [34]. EMT can also occur with sustained NF-κB activation, even in the absence of TGF-β [35]. Ras is a key tyrosine kinase receptor, and activation of the Ras/Akt signaling pathway not only is associated with malignancy in epithelial cells but also can promote EMT [32]. Thereby, highly specific, low-toxicity drugs that are targeted to the EMT-related PI3K/AKT/mTOR pathway must be developed.

Liver kinase B1 (LKB1) has been thought to act as a cancer suppressor [36]. AMPK is the energy sensor and signal transducer and mTOR is a central controller of cell growth and proliferation [37]. LKB1/AMPK activation plays a critical role in stimulating autophagy via decreasing the ratio of p-mTOR/mTOR and p-p70s6k/p70s6k and restraining PI3K/Akt/mTOR activity [38]. In addition, LKB1/AMPK hinders EMT by inhibiting Smad2/3 and TGF-β activity.

GSK-3β is a multifunctional protein kinase that can directly induce autophagy by activating LKB1/AMPK and in turn prohibiting the PI3K/Akt/mTOR pathway [39]. It also indirectly triggers autophagy through promoting the hydrolysis of β-catenin protein, followed by LKB1/AMPK activation and mTOR block. Theoretically, the knockdown of β-catenin can enhance apoptosis and autophagy through activating the LKB1/AMPK pathway and suppressing PI3K/Akt/mTOR signaling in head and neck squamous cell carcinoma [40].

Studies have shown that the LKB1/AMPK/mTORC1 pathway is involved in nesfatin-1/nucleobindin-2 (NUCB-2)–mediated EMT in colon cancer, and ZEB-1 is critical for regulation of NUCB-2-mediated migration and invasion [41]. The activation of LKB1/AMPK inhibits the migration of TGF-β–stimulated cancer cells by inhibiting Smad2/3 activity, which suggests that AMPK may be a target for cancer drug therapy [42].

Ras oncogene is a member of the GTPase gene family. The Ras signaling pathway induces EMT and has dual effects on autophagy. Ras promotes autophagy by Rac1/Mkk7/JNK and Ras/Raf1/MEK1/2/ERK signaling pathway and restrains autophagy by stimulating PI3K/AKT/mTORC1 pathway. Accumulating research has shown that the activated Ras protein will trigger multiple downstream pathways, causing abnormal cell proliferation and tumorigenesis.

Ras has dual effects on autophagy. First, Ras protein mutation does not only activate the Ras/Rac1/Mkk7/JNK pathway (with JNK in turn binding to Atg5/Atg7) [43], but also induces the Ras/Raf1/MEK1/2/ERK signaling pathway. The latter not only improves the transcriptional expression of BNIP and promotes BNIP-induced Bcl-2 release from Beclin1 but also induces the binding of Noxa to Mcl-1 and Beclin-1 dissociation from Mcl-1, which results in autophagy activation [44]. Second, autophagy is inhibited by activating Ras protein, boosting the Ras/PI3K/AKT/mTORC1 pathway and blocking ULK1/Atg13/FIP200 complex formation, which is essential for initiation of autophagy [45]. It is worth noting that a recent article suggested that activated Ras and mutant p53 may synergistically regulate autophagy [46].

Further, a report demonstrated that intracellular PD-L1 prominently activates the EMT process by interacting with H-Ras, which led to Ras/ERK/EMT activation [47]. It is distinct that the Nogo-B receptor (NgBR) is a specific receptor of Nogo-B that regulates vascular remodeling and angiogenesis, which triggers EMT based on the enhancement of EMT-related proteins and SNAIL1 protein expression via activation of the Ras/ERK pathway [48]. What’s more, the TGF-β–activated ERK pathway is necessary to mediate EMT in vitro [49]. Although Ras/Raf/MAPK activation alone cannot lead to prostate cancer initiation, it apparently accelerates progression caused by phosphatase and tensin homolog deleted from chromosome ten (PTEN) loss, accompanied by EMT and metastasis [50].

Currently, several PI3K/AKT/mTOR pathway-targeted drugs have been developed, such as curcumin, FTY720, and Bufalin [51–53]. However, these drugs need to be further improved because of the inevitable toxic side effects. Importantly, the PI3K/AKT/mTOR signaling pathway plays an irreplaceable role in resistance to radiotherapy and chemotherapy. The results have shown that BEZ235 can significantly enhance radiosensitivity by inhibiting the PI3K/AKT/mTOR signaling pathway [54, 55]. In addition, danusertib inhibits the PI3K/AKT/mTOR signaling pathway by inducing the activation of P38 MAPK, resulting in autophagy activation, which suggests that autophagy has a significant inhibitory effect on EMT [56, 57].

### Beclin-1 signaling pathway

**Fig. 2:**
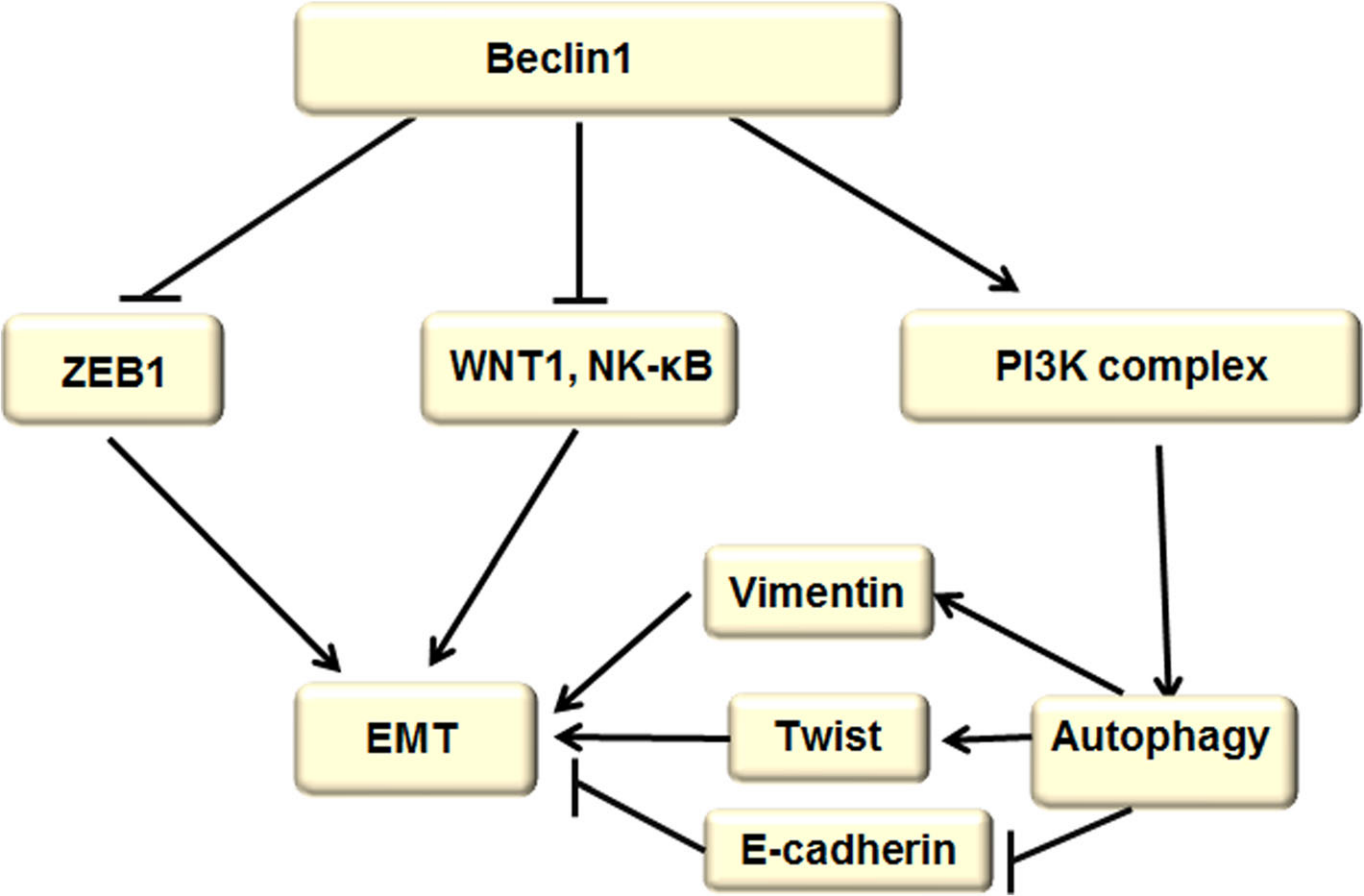
Beclin-1 signaling pathway regulated autophagy and EMT. The Beclin-1 gene triggers autophagy by forming the PI3K complex and prohibiting EMT through down-regulating ZEB1, WNT1, and NF-κB. Additionally, Beclin-1-induced autophagy accelerated EMT by up-regulating vimentin and Twist expression and decreasing E-cadherin expression

Beclin-1 is a homologous gene of the yeast gene Atg6/Vps30, which binds to VPS34 (Catalytic Subunit of ClassIIIPI3K) to construct a complex for inducing autophagy and prohibits EMT through down-regulating ZEB1, WNT1, and NF-κB. Profoundly, Beclin-1-induced autophagy accelerated EMT by up-regulating vimentin and Twist expression and decreasing E-cadherin expression (Fig. 2). It has been used as an independent biomarker for predicting overall survival and progression-free survival in patients with gastric and liver cancer [58].

It was reported that the Beclin-1 gene can trigger autophagy by forming the PI3K complex [59]. A growing body of research has shown that Beclin-1 knockout mice are more prone to spontaneous cancers. Clinic studies have confirmed that the cancer incidence increased after Beclin-1 was suppressed, such as ovarian, breast, and prostate cancers [60]. However, it has also been reported that overexpression of autophagy protein Beclin-1 in mammalian cells can cause cell death [61].

Current studies have shown that knockdown of Beclin-1 causes thyroid cancer cells to lose their epithelial properties and acquire mesenchymal characters consistent with EMT through stabilizing ZEB1 mRNA, and there is a negative correlation between Beclin-1 and ZEB1 in thyroid cancer [62]. In a further study, Beclin-1 gene knockout or low expression is involved in the activation of WNT1 and NF-κB, leading to cancer cell metastasis, which suggests that the knockout or low expression of the Beclin-1 gene may promote EMT and cancerogenesis by activating the WNT1 pathway, resulting in poor prognosis [63, 64]. Surprisingly, knockdown of Beclin-1 by small interfering RNA (siRNA) significantly inhibited the autophagy activation induced by rapamycin, consequentially suppressing EMT and the invasiveness of colon cancer cells via promoting vimentin and Twist down-regulation and E-cadherin up-regulation, suggesting that inhibiting Beclin-1-induced autophagy would an effective anticancer strategy [65].

### P53 signaling pathway

**Fig. 3:**
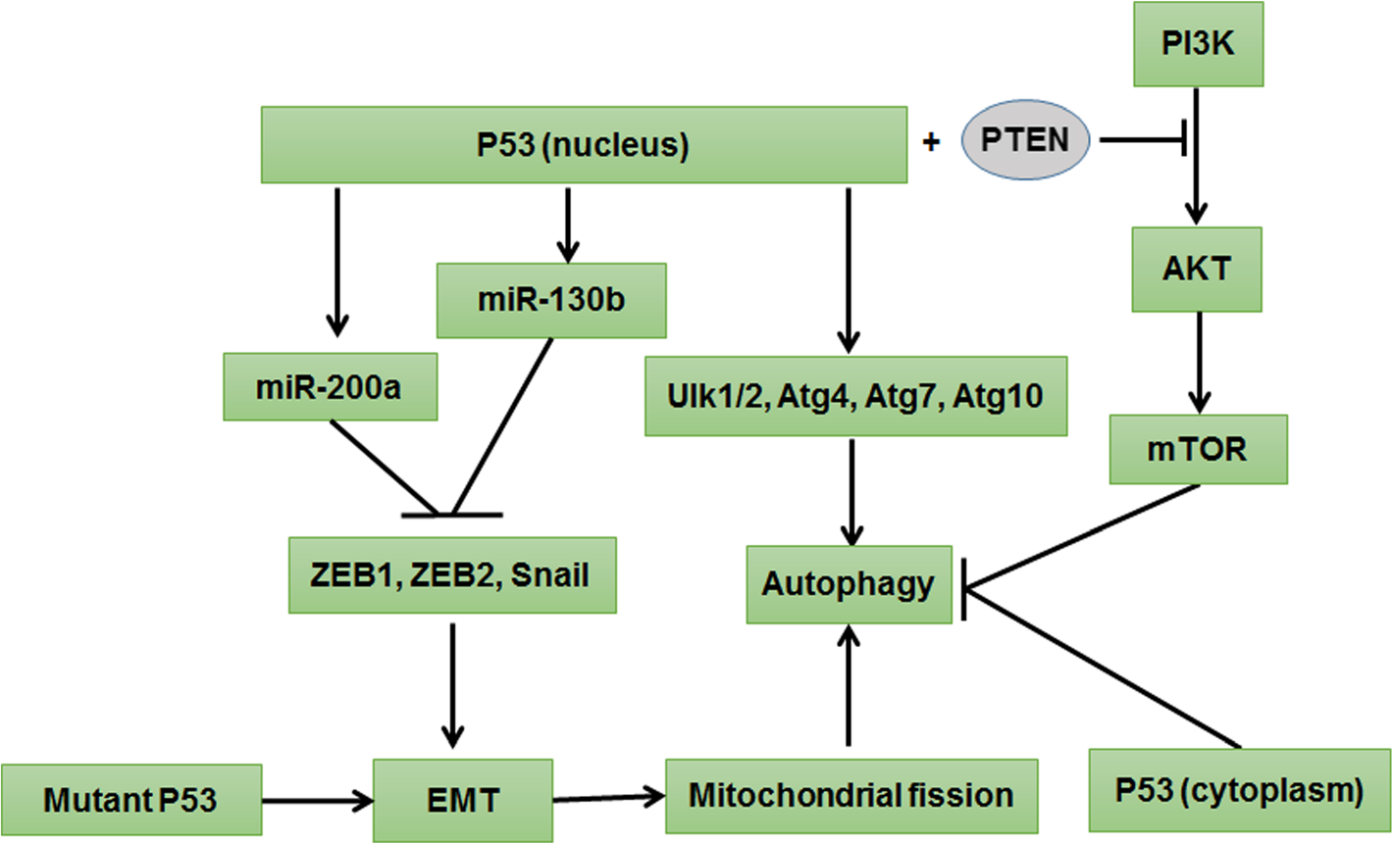
P53 signaling pathway regulated autophagy and EMT. Nucleus P53 promotes the up-regulation of autophagy by down-regulating PI3K/AKT/mTOR signaling and enhancing the expression of autophagy-related genes, including Ulk1/2, Atg4, Atg7, and Atg10. Nevertheless, P53 inhibits autophagy in the cytoplasm. The nucleus P53 reduced the expression of ZEB1, ZEB2, and SNAIL by activating the expression of miR-200a and miR-130b, resulting in EMT inhibition. Significantly, mutant P53 triggers EMT and mitochondrial fission that in turn strengthens autophagy

P53 is a well-known cancer suppressor protein, which mediates cancer inhibition mainly through triggering autophagy dependence on autophagy-related gene expression and PI3K/AKT/mTOR inhibition and by blocking EMT based on decreased expression of ZEB1, ZEB2, and SNAIL. Interestingly, mutant P53 can trigger EMT and mitochondrial fission that in turn promote autophagy [46] (Fig. 3).

In the nucleus, P53 can down-regulate PI3K/AKT/mTOR signaling via interaction with PTEN, which promotes the up-regulation of autophagy. In addition, P53 enhances the expression of autophagy-related genes, including Ulk1/2, Atg4, Atg7, and Atg10 [66]. Increased autophagy continually contributes to p53-dependent apoptosis and cancer suppression. Nevertheless, P53 will inhibit autophagy in the cytoplasm [67], but the specific mechanism remains to be illustrated.

Furthermore, P53 can also simultaneously regulate EMT, and the nucleus P53 can reduce the expression of ZEB1, ZEB2, and SNAIL by activating the relevant microRNA, contributing to EMT inhibition [68, 69]. For instance, in pterygium, inactivation of p53 influences miR-200a expression, resulting in EMT progress through up-regulating ZEB1, ZEB2, and SNAIL gene expression [70]. Mutant P53 can bind to miR-130b promoter and inhibit its transcription, which induces the expression of ZEB1, promotes EMT occurrence, and enhances the ability of cell invasion [71]. Therefore, a targeted therapy strategy of the mutant p53 gene is likely to achieve excellent curative effects. In brief, increased stability and expression of p53 in the nucleus can induce autophagy and inhibit EMT, which suggests a wonderful strategy for anticancer therapy.

### JAK/STAT signaling pathway

**Fig. 4:**
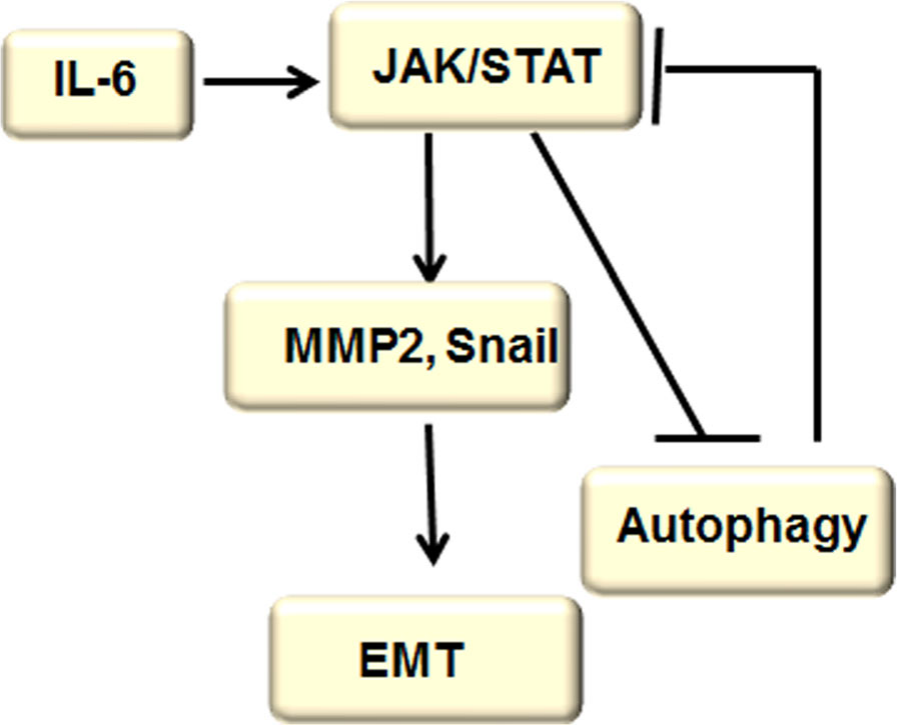
JAK/STAT signaling pathway regulated autophagy and EMT. Activation of JAK/STAT protein is stimulated by IL-6, leading to the up-regulation of the expression of MMP-2 and SNAIL and activation of EMT. On the other hand, the extracellular IL-6–mediated JAK/STAT signaling pathway accelerates the cancer process by prohibiting autophagy. Furthermore, autophagy induction hinders EMT through suppressing JAK/STAT signaling

The JAK/STAT signaling pathway has a significant effect on essential cellular mechanisms such as proliferation, invasion, survival, inflammation, and immunity via inducing EMT and inhibiting autophagy, and autophagy induction hinders EMT through suppressing JAK/STAT signaling. It has been demonstrated that JAK/STAT signaling can transmit extracellular signals to the nucleus by activating receptor tyrosine kinase signaling and transcription activating factor–targeted genes (Fig. 4).

At present, a number of observations about human solid tumors and hematological malignancies have found that JAK/STAT signaling pathway activation is closely involved in cancer cell proliferation, adjacent invasion, and distant metastasis. For example, activation of JAK/STAT protein stimulated by IL-6 up-regulates MMP-2 and SNAIL expression, which results in EMT [72, 73]. However, the JAK2/STAT3 inhibitor WP1066 prevents IL-6–induced activation of the JAK2/STAT3 pathway and EMT [74]. Furthermore, ovatodiolide can efficiently suppress nasopharyngeal cancer development by inducing apoptosis and inhibiting EMT and is consistent with repression of the JAK/STAT signaling pathway [75].

On the other hand, the extracellular IL-6–mediated JAK/STAT signaling pathway accelerates the cancer process by prohibiting autophagy [76]. Recent studies have shown that esveratrol can induce autophagy and hinder ovarian cancer cell migration by inhibiting the IL-6–mediated JAK/STAT signaling pathway. Likewise, quercetin induces autophagy by inhibiting the STAT3 pathway in primary effusion lymphoma [77, 78]. Previously, the report has shown that docetaxel-mediated autophagy significantly decreased castration-resistant prostate cancer (CRPC) cell viability and metastasis by inhibiting STAT3 [79]. Hence, autophagy activators might be used to hinder EMT through suppressing JAK/STAT signaling.

### The integrin signaling pathway

**Fig. 5:**
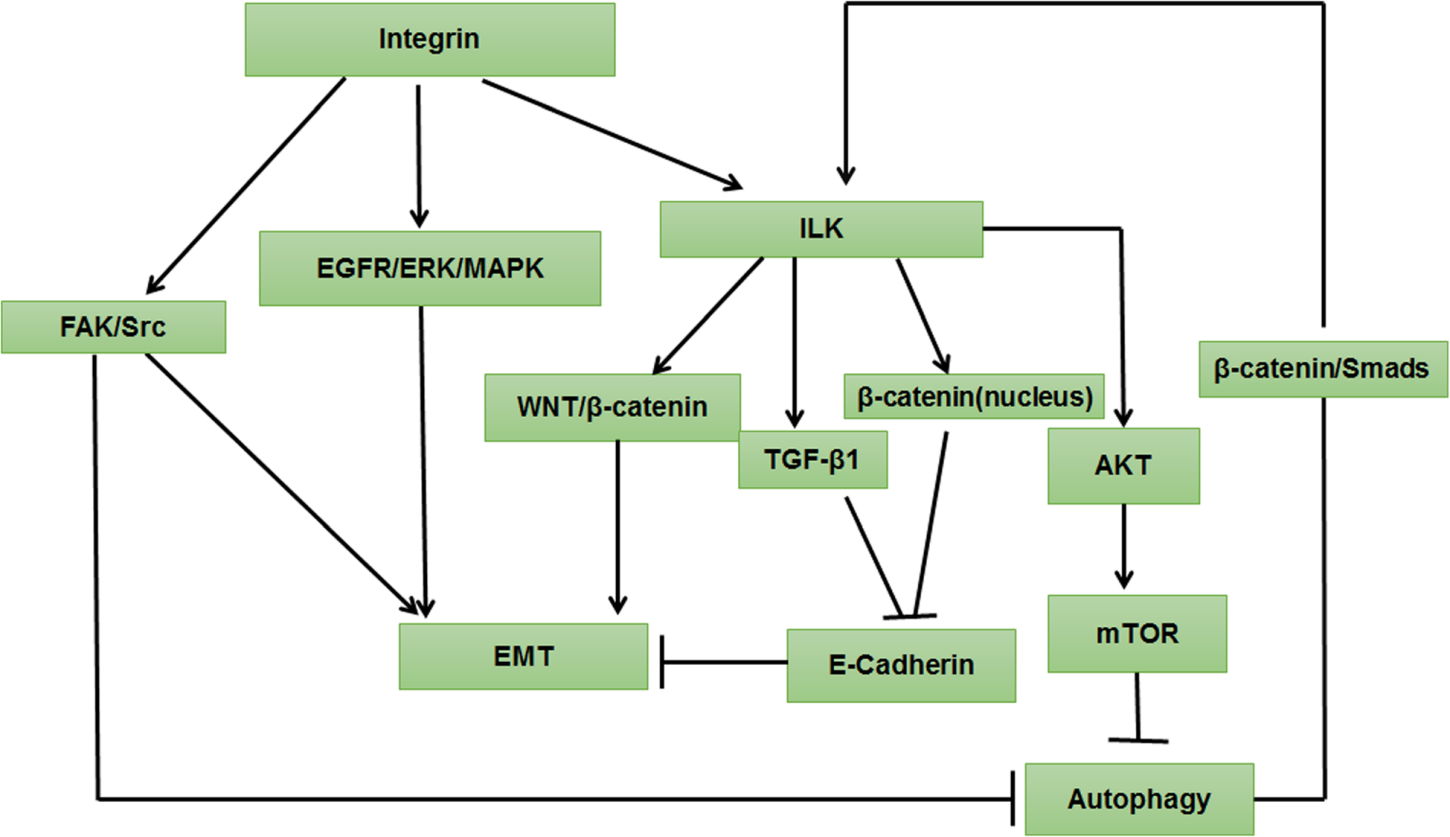
The integrin signaling pathway regulated autophagy and EMT. FAK-Src–mediated integrin pathway has been shown to inhibit autophagy and promote EMT. In addition, integrin enhances EMT by stimulating the EGFR-ERK/MAPK signaling pathway. ILK accelerates EMT by activating the WNT/β-catenin pathway. Similarly, ILK can promote EMT development by transferring β-catenin into the nucleus, causing down-regulation of E-cadherin. Additionally, integrin signaling promotes TGF-β1–dependent down-regulation of E-cadherin expression, which is essential for EMT induction. Furthermore, ILK inhibits autophagy by promoting the phosphorylation of AKT and activating mTOR. By contrast, autophagy stimulates β-catenin and Smad signaling to enhance ILK expression, resulting in EMT promotion

Integrin-mediated signaling pathways have been found to control multiple mechanisms, such as cancer cell survival, proliferation, differentiation, and migration, by modifying the microenvironment. In brief, the integrin pathway inhibits autophagy. The integrin-regulated EMT is mainly mediated by focal adhesion kinase (FAK) and ILK; both FAK-Src–mediated and ILK-mediated integrin pathways induce EMT. Furthermore, autophagy promotes EMT via linking β-catenin and Smad signaling dependent on up-regulation of ILK (Fig. 5).

FAK is a linker molecule that aggregates different signaling proteins. The activation of the integrin-mediated FAK-Src pathway has been shown to inhibit autophagy and promote E-cadherin–dependent collective cell movement and EMT, leading to cancer development. Consequently, knockdown of FAK by siRNA or inhibition of Src kinase activity by dasatinib could inhibit E-cadherin–mediated cell-cell adhesions and EMT [80]. Epidermal growth factor (EGF) can also induce EMT in pancreatic cancer cells via stimulating the integrin/EGFR-ERK/MAPK signaling pathway [81].

The overexpression of ILK can promote EMT development by transferring β-catenin into the nucleus, causing down-regulation of E-cadherin [82]. In addition, integrin signaling promotes TGF-β1–dependent down-regulation of E-cadherin expression, which is essential for EMT induction in RCC. Therefore, the strategies targeted to the integrin–TGF-β1 interplay may represent a therapeutic target in RCC [83]. In addition, ILK acts as a downstream regulator of TGF-β, which accelerates EMT by activating the WNT/β-catenin pathway. Interestingly, TGF-β1 and Β-streptin potentiate the expression of ILK through the β-chain protein/Smad2 signaling pathway in renal fibrosis cells [84].

Furthermore, ILK inhibits autophagy by promoting the phosphorylation of AKT and activating mTOR. Knocking down ILK expression increases autophagy and protects cells from senescence induced by hyperphosphatemia [85]. By contrast, autophagy activity can stimulate β-catenin and Smad signaling by forming the p-β-catenin/p-Smad2 complex to enhance ILK expression, resulting in EMT promotion, indicating that autophagy inhibitors have the great capability to block EMT and cancer development by repressing β-catenin/Smad2 ILK activity [84].

### WNTs signaling pathway

**Fig. 6:**
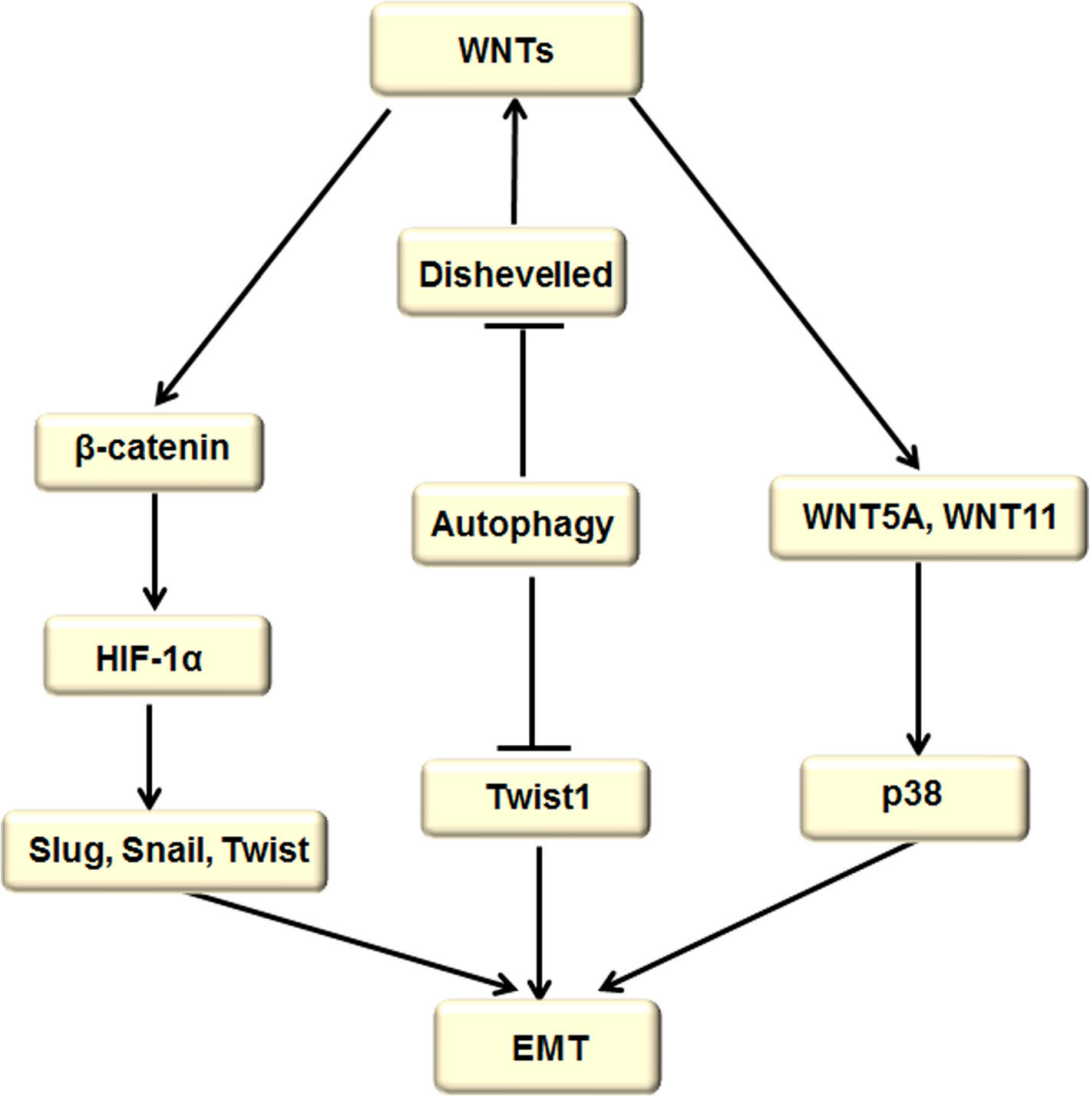
WNTs signaling pathway regulated autophagy and EMT. The WNTs pathway consists of the classical pathway and nonclassical WNT pathway. The classical pathway (WNT/β-catenin signaling pathway) directly leads to HIF-1α activation, which in turn results in overexpression of SLUG, SNAIL, and TWIST and induction of EMT. The nonclassical WNT pathway mainly contains two WNT proteins, WNT5A and WNT11, which facilitate EMT by inducing p38 (Mapk14) phosphorylation. Dishevelled (Dvl) is a basic and central component of WNT signaling, and it plays an important role in both β-catenin–mediated canonical and β-catenin–independent noncanonical WNT signaling. Dvl expression and stability are negatively controlled by autophagy in the late stages of cancer development, which in turn inhibits the WNT process. On the other hand, autophagy can decrease the stability of TWIST1 protein and hinder EMT

The WNTs pathway consists of the classical pathway and nonclassical WNT pathway, which accelerate EMT, and autophagy can down-regulate EMT by degrading the Twist1 protein and inhibiting WNTs pathway. Reduced WNTs signaling is correlated with loss of CSC viability (Fig. 6).

In the nonclassical WNT pathway, two WNT proteins, WNT5A and WNT11, facilitate EMT by inducing p38 (MAPK14) phosphorylation [86]. In the classical pathway, WNT/β-catenin, a typical WNT signaling pathway, directly leads to HIF-1α–induced EMT by combining with the intracellular domain of E-cadherin, subsequently connecting to the actin cytoskeleton and mediating intercellular adhesion. Here, we have shown that hypoxia or overexpression of HIF-1α promotes EMT and contributes to metastatic phenotypes. On the one side, HIF-1α up-regulates the expression of TWIST by directly binding to the hypoxia-response element in the TWIST proximal promoter [87]. On the other side, HIF-1α also results in overexpression of SLUG and SNAIL and the formation of EMT [88]. As for EGF-induced EMT, we focused on transcription repressors of E-cadherin, TWIST, SLUG, and SNAIL; the results showed that cancers express high levels of TWIST, which can be further enhanced by EGF [89].

There are cross-points between the WNT signaling pathway and the TGF-β, PI3K/AKt pathway. One report proved that osteopontin promotes the progression of hepatocellular carcinoma (HCC) via stimulating the PI3K/AKT/Twist signaling pathway, contributing to the enhancement of EMT [90]. Similarly, the TGF-β–induced ERK/MAPK pathway contributes to EMT induction, since ERK is required for removing cell adherens junctions to increase cell mobility [91].

Recently, we found that autophagy deficiency stabilizes the TWIST1 protein through accumulation of SQSTM1/p62. Mechanically, the Twist1 degradation is blocked by the interaction between SQSTM1 and TWIST1 in autophagosomes and proteasomes. Twist1 is a key downstream regulator of p62, which suggests that targeted p62-mediated TWIST1 stabilization is a promising therapeutic strategy for cancer prevention and treatment [92, 93]. In colon cancer cell lines, SNAIL overexpression increases the expression of the WNT signal target gene. The mechanism is that the interaction between SNAIL-N and β-catenin further activates the expression of the WNT downstream target gene, resulting in positive feedback of WNT signaling.

In eukaryotic cells, autophagy is a highly conserved self-digestive process that can produce nutritional substance, relieve metabolic stresses, and maintain cell survival. Dishevelled (Dvl) is a basic and central component of WNT signaling that plays an important role in both β-catenin–mediated canonical and β-catenin–independent noncanonical WNT signaling [94]. Dvl expression and stability are negatively controlled by autophagy in the late stages of cancer development, which in turn inhibits the WNT process [95, 96]. In a further study, the WNT signaling antagonist Dapper1 was induced by autophagy-accelerated Dvl2 degradation [97], which is mediated by GABA(A) receptor–associated protein like 1 (GABARAPL1), a cancer repressor, and p62 is required for the interaction of Dvl2 and GABARAPL1. GABARAPL1-mediated Dvl2 degradation is blocked when administered with 3-MA, a specific inhibitor of autophagy [98]. In addition, GABARAP is a cytoplasmic cadherin-6 (CDH6) conjugate. CDH6 is a type 2 cadherin, which drives EMT [99]. Previous studies have confirmed that the silence of CDH6 reverses EMT and reduces thyroid cancer cell metastasis, accompanied by autophagy induction [98, 100]. Therefore, the interplay between the degradation of Dvl, inhibition of WNT signal, and induction of autophagy provides a promising approach for anticancer treatment.

### NF-κB signaling pathway

**Fig. 7:**
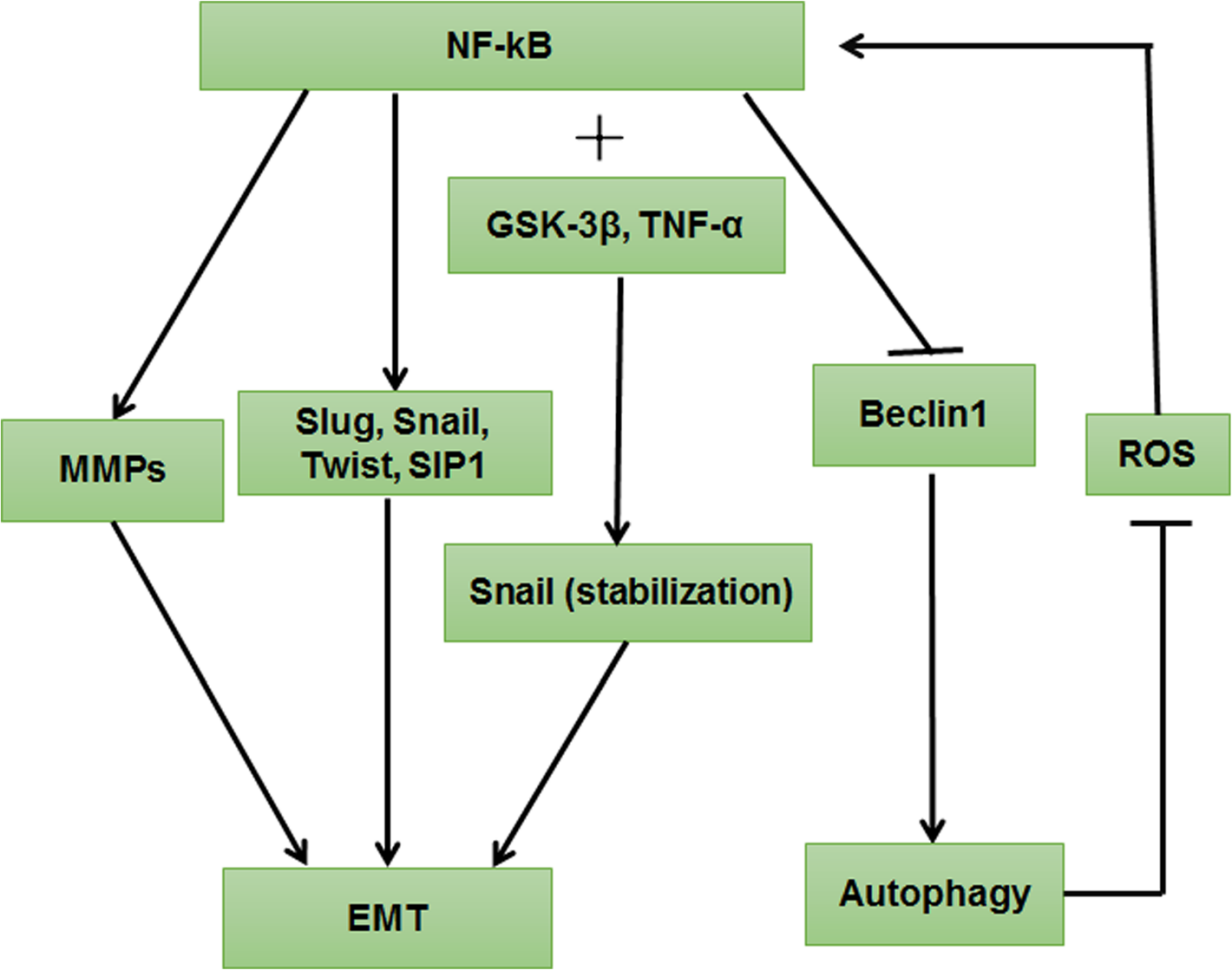
NF-κB signaling pathway regulated autophagy and EMT. NF-κB activation increased transcriptional activation of EMT regulator genes expression via binding directly to the sites of EMT transcription factors, including SNAIL1, SLUG, TWIST1, and SIP1 promoter. In addition, TNF-α–mediated stability of SNAIL protein is strengthened by GSK3β activity, which is dependent on NF-κB activation. Furthermore, NF-κB binding to MMPs promotes SNAIL transcription. NF-κB can down-regulate autophagy by inhibiting Beclin-1, an initiator of autophagy. However, autophagy activation suppresses ROS–NF-κB signaling to down-regulate MMPs expression, contributing to EMT inhibition

NF-κB is another important regulator of EMT. Its activation has been associated with aggressiveness and the metastatic potential of carcinomas [101], which inhibits autophagy by down-regulating Beclin-1 and promotes EMT by up-regulating related EMT markers. However, autophagy could suppress NF-κB signaling to inhibit EMT by down-regulating MMPs expression (Fig. 7).

In human breast cancer, NF-κB activation increased transcriptional activation of EMT regulator gene expression by binding directly to the sites of EMT transcription factors, including SNAIL1, SLUG, TWIST1 and SIP1 promoter, which promotes an aggressive phenotype of breast cancer cells [102]. Similarly, a study found that uric acid induces EMT in renal tubular epithelial cells by the activation of the TLR4/NF-κB signaling pathway [103]. In addition, NF-κB can regulate SNAIL-induced and ROS-dependent signaling pathways in response to MMP-3. The mechanism is that MMP-3 binding to p65 and cRel NF-κB subunits promotes SNAIL transcription. Specifically, MMPs are involved in several pathways that induce EMT, which provide a potential treatment for cancer [104]. Furthermore, tumor necrosis factor–α (TNF-α)–mediated stability of the SNAIL protein is strengthened by GSK3β activity, which is dependent on NF-κB activation [105].

NF-κB can stimulate or hinder autophagy by different mechanisms. Researchers have shown that NF-κB can down-regulate autophagy by inhibiting Beclin-1, an initiator of autophagy [106]. Notably, blocking NF-κB can significantly inhibit the proliferation of hepatocarcinoma cells, which is associated with autophagy enhancement [107]. On the other hand, ROS has an effect on cell transformation, metastasis, and response to therapy at different stages of cancer development, which stimulates NF-κB–dependent autophagy [105]. Inhibition of ROS–NF-κB–dependent autophagy could enhance brazilin-induced apoptosis in head and neck squamous cell carcinoma [108]. However, autophagy activation could suppress ROS–NF-κB signaling to down-regulate MMP-2 and MMP-9 expression, contributing to EMT inhibition [109]. Thus, autophagy activators might be used to prohibit NF-κB signaling, consequently repressing EMT and cancer progression.

### TGF-β signaling pathway

**Fig. 8:**
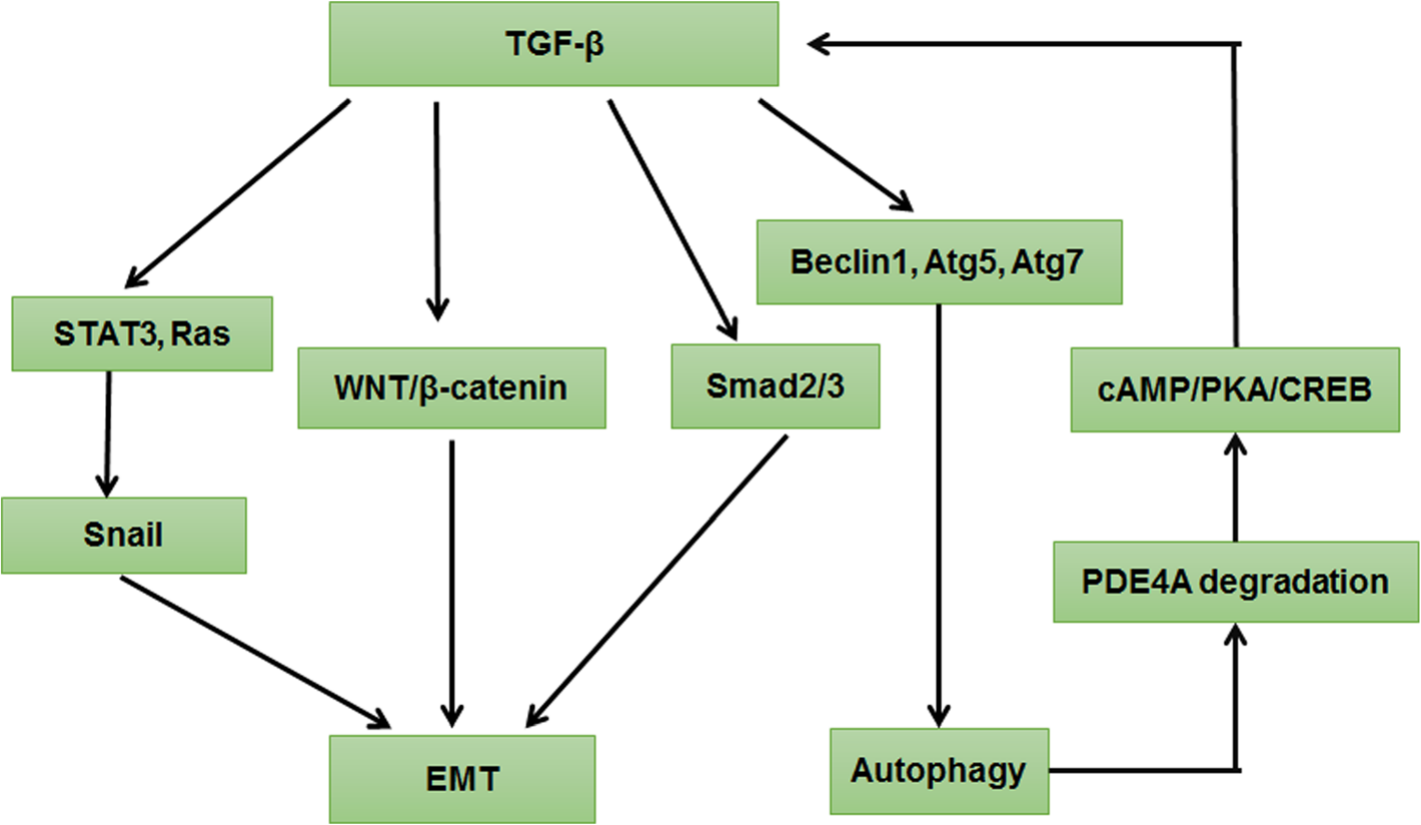
TGF-β signaling pathway regulated autophagy and EMT. Activation of TGF-β/Smad3 in epithelial cells triggers EMT, and the activation of TGF-β/Smad2 signaling pathway also stimulates EMT. Moreover, TGF-β1 induces EMT and cancer metastasis by directly targeting the cytoplasmic domain of E-cadherin (CDH1) and activating WNT/β-catenin signaling. Sometimes, TGF-β cooperates with synergistic factors to induce EMT, such as Ras. Once TGF-β is stimulated, EMT-related transcription factor STAT3 interacts with Ras, which induces SNAIL expression and promotes EMT. Naturally, TGF-β can stimulate the expression of mRNA transcripts of several autophagy-related genes, such as Beclin-1, Atg5, Atg7, and death-associated protein kinase (Dapk), and it induces accumulation of autophagosomes and activation of autophagic flux, which potentiates the induction of the autophagy. It is worthy that autophagy induces TGF-β1 expression and TGF-β1-dependent EMT via triggering cAMP/PKA/CREB signaling, which relies on autophagy-dependent phosphodiesterase 4A (PDE4A) degradation

TGF-β, secreted by cancer cells and stromal fibroblasts in the cancer microenvironment, is considered as a primary inducer of EMT through inducing SNAIL expression and cooperating with Smad2 and Smad3 and WNT/β-catenin signaling [110]. In addition, TGF-β triggers autophagy by stimulating expression of mRNA transcripts of several autophagy-related genes. Notably, autophagy enhances TGF-β1 expression by inducing activation of cyclic adenosine monophosphate (cAMP)/ protein kinase A (PKA)/cAMP response element binding (CREB) signalling, leading to EMT progression (Fig. 8).

The TGF-β pathway contributes to EMT occurrence by mediating Smad and non-Smad signal transduction pathways. Activation of TGF-β/Smad3 in epithelial cells triggers significant morphological and functional phenotypical alterations [111], which indicates that TGF-β/Smad3–dependent signaling plays a key role in regulating autophagy-induced EMT [112]. In addition, M2 macrophages also induce EMT through stimulating the TGF-β/Smad2 signaling pathway [113]. Sometimes TGF-β cooperates with synergistic factors to induce EMT, such as Ras and β-catenin. Once TGF-β is stimulated, EMT-related transcription factor STAT3 interacts with Ras, which induces SNAIL expression and promotes EMT [114, 115]. Profoundly, miR-23a promotes TGF-β1–induced EMT and cancer metastasis in breast cancer cells by directly targeting the cytoplasmic domain of E-cadherin (CDH1) and activating WNT/β-catenin signaling [116]. More importantly, TGF-β1 has been identified as the most effective factor that can independently induce EMT [117].

Among these, TGF-β has dual effects on cancer occurrence and development, which depends on the cell type and environment [108]. First, TGF-β1 promotes human carcinoma cell invasion by inducing autophagy, and the autophagy inhibitor 3-MA could effectively reverse this process [118]. Second, TGF-β stimulates the expression of mRNA transcripts of several autophagy-related genes, such as Beclin-1, Atg5, Atg7, and death-associated protein kinase (Dapk), and it induces accumulation of autophagosomes and activation of autophagic flux, which potentiates the induction of the proapoptotic Bcl-2 family protein Bim and contributes to Bim-mediated apoptosis in hematopoietic cells [119, 120]. It is worthy that autophagy induces TGF-β1-dependent EMT in HCC via triggering cAMP/PKA/CREB signaling, which relies on autophagy-dependent phosphodiesterase 4A (PDE4A) degradation [121]. Hence, further researches could focus on developing the autophagy inhibitors to down-regulate PDE4A-activated cAMP/PKA/CREB signaling and TGF-β1 induced EMT.

### Interplay between cytoskeleton and mitochondria

**Fig. 9:**
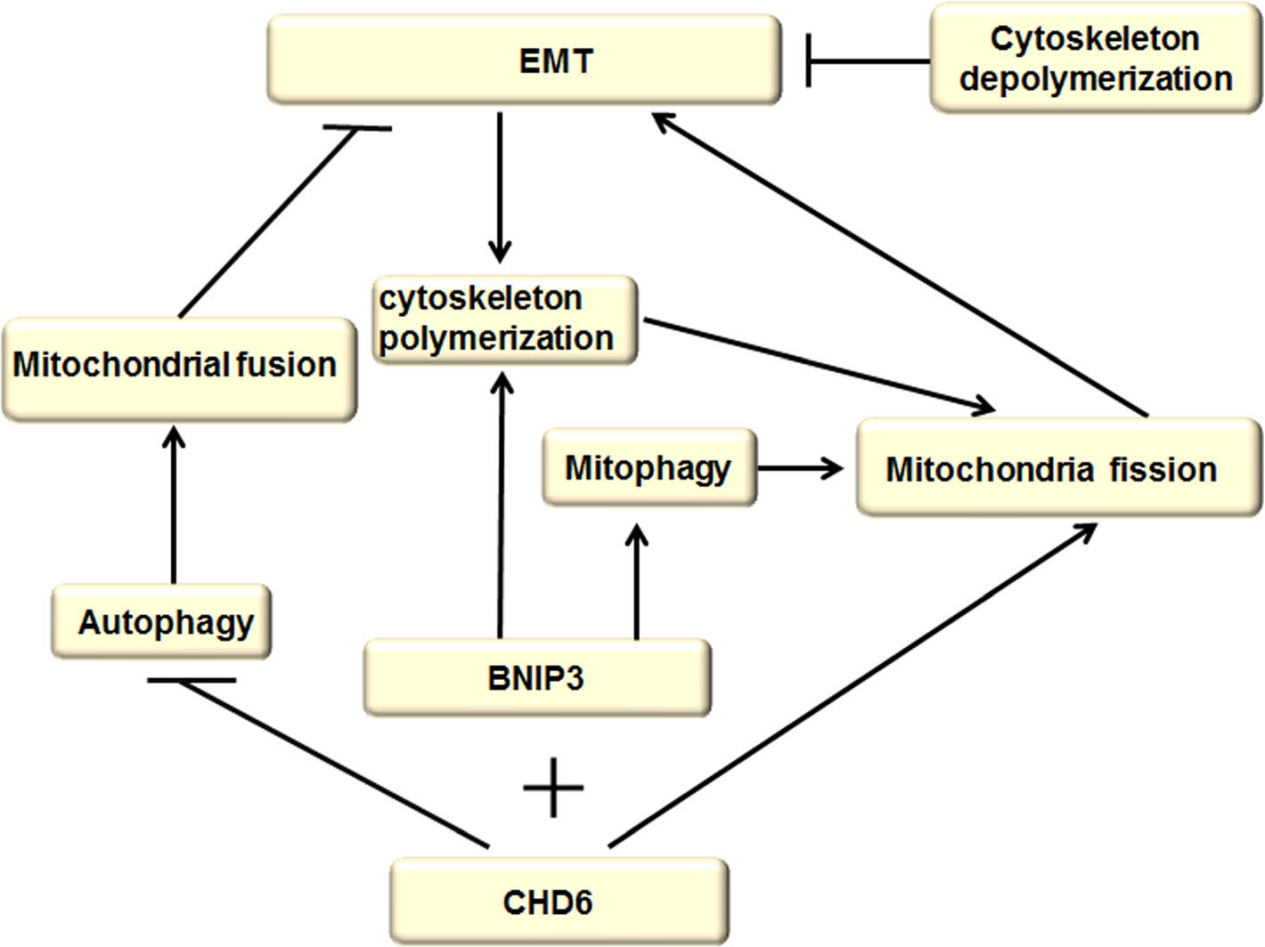
Interplay between cytoskeleton and mitochondria. Cytoskeleton polymerization induced by EMT, which in turn supports mitochondrial fission that are essential for further sustain EMT process by providing energy supplies, and depolymerization of actin cytoskeleton is sufficient for reversing EMT phenotype. Massive activation of autophagy induces mitochondrial fusion and the reconstitution of mitochondrial network, which subsequently reduces the number of available free mitochondria and counteracts EMT. Mitochondrial protein BNIP3 potentially supported mitochondrial fission and turnover through stimulating mitophagy by directly binding to both mitochondria and the autophagosomal protein LC3, but also enhanced cytoskeleton polymerization. The interaction between BNIP3 and cadherin-6 (CDH6) drives EMT, restrains autophagy and promotes mitochondrial fission

The cytoskeleton structures are essential for promoting cell movement and cytoskeleton remodeling, especially supporting EMT activation. Mitochondria are considerably multifunctional organelles, which prominently mediate energy conversion and are crucial regulators of a number of signaling pathways associated with cancer progression. In particular, the interaction of mitochondria and cytoskeleton plays a critical role in regulating autophagy and EMT (Fig. 9).

Accumulating evidence has indicated that actin cytoskeleton remodeling potentially drives EMT, invasion and metastasis [122]. Mechanically, hypoxia-induced phosphatidylinositol (4,5) bisphosphate (PIP2) level increased in cancer cells by activating the HIF-1α/RhoA/ROCK1 signaling pathway supported actin filament (F-actin) expression and attenuate the binding amounts of F-actin and capping actin protein of muscle Z-line alpha subunit 1 (CAPZA1), which in turn enhanced EMT [123, 124]. In addition, actin dynamics and membrane-cytoskeleton scaffolds are required for the early formation of autophagosome in starvation induced autophagy [125]. It was reported that the colocalization of actin filaments with important autophagy markers [126]. Mitochondria are the important energy resource for multiple biological metabolisms, such as autophagy, migration and invasiveness. Increased fission and loss of mitochondrial network have been identified as characteristics of oncogenic transformation and accelerates EMT and cancer migration [127, 128].

Notably, mitochondrial dynamics provide ATP for cytoskeleton remodeling to promote EMT during cancer progression, while autophagy regulates mitochondrial dynamics by eliminating the damaged mitochondria. The cytoskeleton is composed of three main types of polymers: actin filaments, microtubules and intermediate filaments, which have been associated with mitochondrial network properties and various mitochondrial functions [129]. For instance, cytoskeleton polymerization and remodeling induced by EMT, which in turn supports mitochondrial fission that are essential for further sustaining cell migration and EMT process by providing energy supplies, and depolymerization of actin cytoskeleton is sufficient for reversing EMT phenotype [129, 130]. Further findings have demonstrated that massive activation of autophagy induces mitochondrial fusion and the reconstitution of mitochondrial network, which subsequently reduces the number of available free mitochondria and counteracts cell migration and EMT [130]. Recently, it was reported that the mitochondrial protein, B-cell lymphoma 2 (BCL-2) interacting protein 3 (BNIP3) in melanoma potentially supported mitochondrial fission and turnover through stimulating mitophagy by directly binding to both mitochondria and the autophagosomal protein LC3, but also maintained cellular architecture and enhanced cytoskeleton polymerization [131], which revealed the pro-tumorigenic role of BNIP3 in driving EMT and melanoma cell’s migration. Indeed, BNIP3-silenced melanoma cells resulted in re-organization of focal adhesion sites and repressed cell–cell interaction [132]. Profoundly, the interaction between BNIP3 and CDH6 drives EMT, restrains autophagy and promotes mitochondrial fission through dynamin-related protein 1 (DRP1)-mediated mechanism [99]. Therefore, novel avenues enhancing autophagy to suppress mitochondrial fission and cytoskeleton polymerization have represented a promising strategy for anticancer therapy by prohibiting EMT.

## Regulating EMT by targeting autophagy

**Fig. 10:**
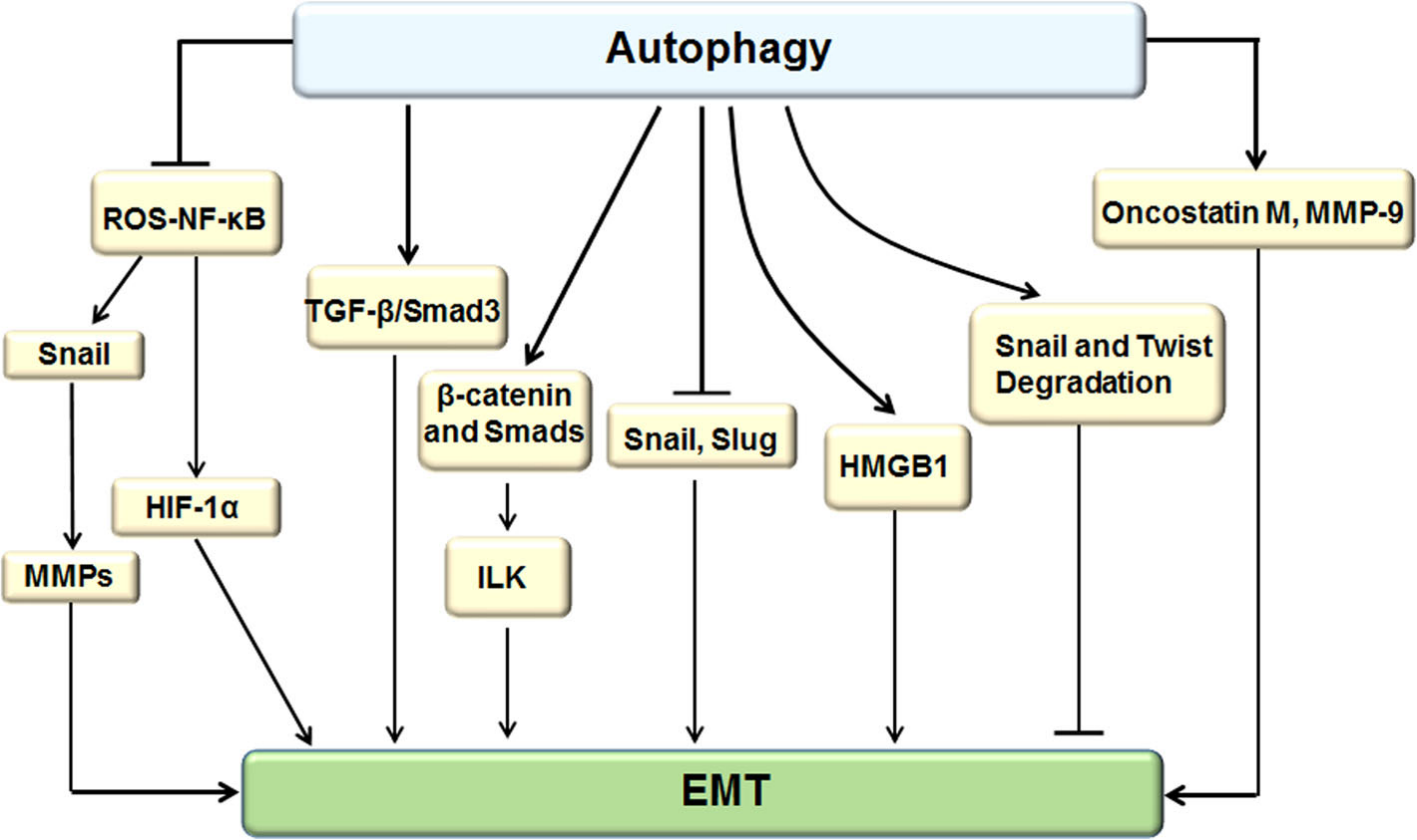
Regulating EMT by targeting autophagy. The autophagy attenuates EMT by inhibiting the overexpression of SNAIL and SLUG and activation of ROS-NF-κB-HIF-1α pathway. Moreover, ROS-induced NF-κB activation up-regulates SNAIL and MMPs expression to promote EMT. Additionally, autophagy accelerates lysosomal- mediated degradation of SNAIL and TWIST, resulting in EMT inhibition. On the other side, autophagy activation enhances EMT by increasing the expression of HMGB1, metastasis-associated protein oncostatin M and MMP-9, facilitating EMT markers expression in both RNA and protein levels accompany with promotion of TGF-β2/Smad signaling pathway activity, and up-regulating ILK by linking β-catenin and Smad signaling to induce EMT

Autophagy and EMT act as major biological processes in the occurrence and development of the cancer. Recent observations indicate that there is a complex link between the two processes. On the one hand, cells are dependent on autophagy activation to survive during the EMT. On the other hand, autophagy functions as the tumor-suppressive signal, which hinders the early phases of metastasis and activation of the EMT [130]. Therefore, regulating EMT by targeting autophagy is a very promising strategy for treating cancer (Fig. 10).

It was reported that inhibition of autophagy may promote EMT through the ROS/heme oxygenase-1 (HO-1) pathway in ovarian cancer. N-acetylcysteine (NAC; ROS scavenging agent) and Znpp (HO-1 inhibitor) could impair the migration and invasion through decreasing the expression of HO-1 and reversing EMT [133]. Another report showed that an autophagy defect can promote cancer cell migration and EMT, enhance aerobic glycolysis, and convert cell phenotype toward malignant, which depends on the activation of the ROS–NF-κB–HIF-1α pathway. EMT and metastasis will be attenuated when ROS is cleaned by the antioxidant NAC [134]. What’s more, MMPs could promote EMT in mammary epithelial cells, which are stimulated by ROS-induced NF-κB–dependent activation of SNAIL [104]. In addition, Myriam Catalano et al. demonstrated that Beclin-1 silence and ATG7 down-regulation could enhance the EMT process by overexpression of SNAIL and SLUG in glioblastoma cells, and cell migration and invasion would be attenuated when autophagy is induced upon starvation and treatment with mTOR inhibitors [7]. Moreover, studies about breast and colon cancers have described that the death effector domain-containing DNA-binding protein (DEDD) not only significantly represses EMT by inducing autophagy through direct interaction with the class III PI-3 kinase (PI3KC3)/Beclin-1 but also promotes the autophagy-mediated lysosomal degradation of SNAIL and TWIST [135, 136]. Accordingly, autophagy exerts pro-survival functions by prohibiting EMT, and autophagy activators represent a potential strategy for therapeutic interventions.

However, autophagy helps cancer cells to survive in stressful environmental and intrinsic conditions and also exerts pro-death functions via heightening EMT, which depends on cell types and the stages of cancer [137]. For instance, high mobility group box 1 (HMGB1) induces EMT in association with increased autophagy through increasing the expression of discoidin domain receptor 1 (DDR1) and decreasing the phosphorylation of mTOR [138]. Moreover, autophagy is critical for activation of TGF-β/Smad3–dependent signaling, leading to EMT and cancer cell invasion in HCC [112]. Besides, TGF-β1–induced autophagy promotes EMT based on up-regulation of ILK by linking β-catenin and Smad signaling [84]. Significantly, Li et al. found that neutrophil autophagy activity can increase the expression of metastasis-associated protein oncostatin M and MMP-9 and contribute to cancer cell metastasis in HCC [139]. In the late development of cancer progression, cell motility and migration capacity is weakened by inhibiting autophagy-related genes, and cell invasion is restored by the activation of autophagy-related genes [140]. It is acknowledged that TGF-β2 could up-regulate autophagy and facilitate EMT marker expression in both RNA and protein levels accompanied by Smad signaling pathway activity. A further study observed that proinflammatory cytokines such as TNF-α, it can down-regulate autophagy and increase ROS levels, thus antagonizing TGF-β2–induced EMT, which suggests that autophagy plays a prometastatic role in facilitating EMT by regulating ROS levels and TNF-α can inhibit EMT by suppressing autophagy [141]. Consequently, these findings may provide novel avenues for therapeutic research by hindering autophagy, which may be beneficial to patients with cancer.

Cancer treatment has been complicated, which is mainly associated with autophagy and EMT. Therefore, inhibition of EMT by controlling autophagy must be beneficial for cancer treatment. However, the relevant therapeutic measures must depend on tissue/cell types (epithelial cell or interstitial cells) and the stages of cancer development (early or advanced); thus, the choice of inductors or inhibitors of autophagy need to be further analyzed.

## Conclusion

Cell autophagy and EMT, which play indispensable and significant roles in the occurrence and development of cancer, bring great challenges to anticancer treatment. Based on the complicated link between autophagy and EMT, therapeutic avenues targeting autophagy have attracted lots of attention by hindering EMT and further suppressing cancer development. Hence, future studies should focus on profoundly exploring the regulation mechanisms between autophagy and EMT at the molecular and genetic levels. In addition, the discovery of various transcription factors that induce EMT and new EMT markers is urgently needed, as they will be beneficial for further understanding the mechanism of EMT and obtaining effective anticancer treatment strategies by blocking metastasis. It has been reported that autophagy has dual effects on EMT depending on the contextual need of cancer cells during metastases. Currently, autophagy inhibitors, such as chloroquine and 3-methyladenine, and autophagy activators, such as rapamycin, have translational applications in anticancer therapy by regulating EMT (Tables 1 and 2). However, there are few observations about the clinical application of autophagy regulators because of the inevitable side effects, such as high cytotoxicity and low specificity. Therefore, we should try our best to discover more potential and accurate approaches to stimulate or block autophagy, subsequently suppressing EMT and controlling cancer development, which might be a more promising approach for anticancer therapy when combined with other anticancer strategies.

**Table 1:** The translational application of autophagy inhibitors in anticancer therapy by regulating EMT

Autophagy Inhibitors	Impair/Enhance EMT	Mechanisms	Cancer Types	References
Chloroquine(CQ)	Impair	Reversing TGF-β-induced EMT	Renal cell carcinoma(RCC)	[6]
CQ	Impair	Down-regulating expression of vimentin and up-regulating expression of E-cadherin	Nasopharyngeal carcinoma (NPC)	[142]
CQ or 3-methyladenine (3-MA)	Impair	Inhibiting TGF-β1/Smad3-induced EMT	Bladder cancer.	[143]
3-MA	Enhance	Attenuating inhibitive effects of miR-16 mimics on TGF-β1-induced EMT	Non-small cell lung carcinoma (NSCLC)	[144]
3-MA	Enhance	Abolishing the sinomenine hydrochloride (SH) -mediated inhibition of vimentin, Snail and Slug expression	Glioblastoma	[145]

**Table 2:** The translational application of autophagy activators in anticancer therapy by regulating EMT

Autophagy Activators	Impair/Enhance EMT	Mechanisms	Cancer Types	References
Rapamycin	Impair	Prohibiting TGF-β-induced EMT	Gallbladder cancer (GBC)	[27]
Rapamycin	Impair	Inhibiting depletion of FBXW7-induced EMT and stem cell-like behavior	Colon cancer	[146]
Rapamycin	Impair	Prohibiting TGF-β-induced EMT	A cell culture model of TGF-β-induced EMT	[147]
Brusatol	Impair	Decreasing expression of N-cadherin, Vimentin and up-regulating expression of E-cadherin	Hepatocellular carcinoma (HCC)	[148]
Combination of P38 inhibitor SB202190 and JUN inhibitor SP600125	Impair	Inhibiting EMT markers expression	Ovarian cancer	[149]
Alisertib (ALS, MLN8237)	Impair	Regulating E-cadherin suppressor with the involvement of Sirt1	Osteosarcoma (OS)	[150]
mTORC inhibitor AZD2014	Impair	Down-regulating the expression of Snail and MMP2	Hepatocellular carcinoma (HCC)	[151]
Metformin	Impair	Inhibit mTOR pathway and regulate expression of the EMT-related markers E-cadherin, N-cadherin, and Snail.	Thyroid cancer	[28]

## Abbreviations

4E-BPs: Eukaryotic initiation factor 4E (eIF4E)-binding proteins
AMPK: Adenosine monophosphate-activated protein kinase
aPKC-ι: Atypical protein kinase C-iota
ATG: Autophagy-related genes
BNIP3: B-cell lymphoma 2 (BCL-2) interacting protein 3
CDH1: Cytoplasmic domain of E-cadherin
CDH6: Cadherin-6
CDH6: Cadherin-6
CQ: Chloroquine
CSC: Cancer stem cell
DDR1: Discoidin domain receptor 1
DEDD: Domain-containing DNA-binding protein
DRP1: Dynamin-related protein 1
EMT: Epithelial-mesenchymal transition
EMT-TFs: EMT-transcription factors
ERK: Extracellular signal-regulated protein kinase
Erα: Estrogen receptor-α
ESRP1: Epithelial splicing regulatory protein 1
FAK: Focal adhesion kinase
FGF: Fibroblast growth factor
FGFR: Fibroblast growth factor receptor
GABARAPL1: GABA(A) receptor-associated protein like 1
GSK3β: Glycogen synthase kinase 3β
HGF: Hepatocyte growth factor
HMGB1: High mobility group box 1
HO-1: Heme oxygenase-1
HRE: Hypoxia-response element
IL: Interleukin
ILK: Integrin-linked kinase
JNK: c-Jun NH2-terminal kinase
LKB1: Liver kinase B1
MMP: Matrix metalloproteinase
mTOR: Mammalian target of rapamycin
NAC: N-acetylcysteine
NF-kB: Nuclear transcription factor kB
NgBR: Nogo-B receptor
P70S6: Phosphorylated ribosomal protein S6
PAS: Pre-autophagosomal structure
PDGFR: Platelet-derived growth factor receptor
PI3K: Phosphatidylinositol 3-kinase
PI3KC3: Class III PI-3-kinase
PKB/AKt: Protein Kinase B
PTEN: Phosphatase and tensin homolog deleted from chromosome ten
ROS: Reactive oxygen species
Sp1: Specificity protein 1
STAT: Signal transducer and activator of transcription
TGF-β: Members of transforming growth factor-β
TNF-α: Tumor Necrosis Factor-α
VMP1: Vacuole membrane protein 1
WSP1: Water stress proteins
ZEB1: Zinc-finger E-box binding homeobox 1

## Authors’ information

As mentioned in the cover page.
